# RuO_2_ Catalysts for Electrocatalytic Oxygen Evolution in Acidic Media: Mechanism, Activity Promotion Strategy and Research Progress

**DOI:** 10.3390/molecules29020537

**Published:** 2024-01-22

**Authors:** Jirong Bai, Wangkai Zhou, Jinnan Xu, Pin Zhou, Yaoyao Deng, Mei Xiang, Dongsheng Xiang, Yaqiong Su

**Affiliations:** 1 Research Center of Secondary Resources and Environment, School of Chemical Engineering and Materials, Changzhou Institute of Technology, Changzhou 213022, China; baijr@czu.cn (J.B.); zhoup@czu.cn (P.Z.); dengyy@czu.cn (Y.D.); xiangm@czu.cn (M.X.); 2 School of Chemistry and Environmental Engineering, Jiangsu University of Technology, Changzhou 213001, China; zhouwangkai1108@126.com (W.Z.); jinnan_xu@126.com (J.X.); 3 School of Medicine and Health, Yancheng Polytechnic College, Yancheng 224005, China; 4 School of Chemistry, Engineering Research Center of Energy Storage Materials and Devices of Ministry of Education, National Innovation Platform (Center) for Industry-Education Integration of Energy Storage Technology, Xi’an Jiaotong University, Xi’an 710049, China

**Keywords:** RuO_2_ catalyst, activity promotion strategy, oxygen evolution reaction, electrocatalysts, acidic media

## Abstract

Proton Exchange Membrane Water Electrolysis (PEMWE) under acidic conditions outperforms alkaline water electrolysis in terms of less resistance loss, higher current density, and higher produced hydrogen purity, which make it more economical in long-term applications. However, the efficiency of PEMWE is severely limited by the slow kinetics of anodic oxygen evolution reaction (OER), poor catalyst stability, and high cost. Therefore, researchers in the past decade have made great efforts to explore cheap, efficient, and stable electrode materials. Among them, the RuO_2_ electrocatalyst has been proved to be a major promising alternative to Ir-based catalysts and the most promising OER catalyst owing to its excellent electrocatalytic activity and high pH adaptability. In this review, we elaborate two reaction mechanisms of OER (lattice oxygen mechanism and adsorbate evolution mechanism), comprehensively summarize and discuss the recently reported RuO_2_-based OER electrocatalysts under acidic conditions, and propose many advanced modification strategies to further improve the activity and stability of RuO_2_-based electrocatalytic OER. Finally, we provide suggestions for overcoming the challenges faced by RuO_2_ electrocatalysts in practical applications and make prospects for future research. This review provides perspectives and guidance for the rational design of highly active and stable acidic OER electrocatalysts based on PEMWE.

## 1. Introduction

Depletion of fossil fuels and the resulting greenhouse gas emissions have significantly increased global energy demands, causing serious environmental problems. To address these challenges, people are increasingly turning to alternative and sustainable energy sources, including hydrogen, an ideal energy carrier characterized by zero carbon dioxide emissions and high energy density [1,2]. Electrocatalytic water splitting as a potential means of sustainable hydrogen production depends largely on the development of high performance catalysts [3,4,5]. The oxygen evolution reaction (OER) is a slow four-electron transfer process with a high overpotential and significant energy loss, resulting in slow kinetics and practical voltage exceeding theoretical values. Therefore, efficient, stable and inexpensive electrocatalysts are essential to improve the efficiency of water splitting under acidic and alkaline conditions for sustainable hydrogen production. Proton Exchange Membrane Water Electrolysis (PEMWE) have developed rapidly, with lower resistance loss and higher current density compared to alkaline electrolyte. This means more hydrogen can be produced per unit time, and the produced H_2_ has high purity, which are beneficial for improving energy efficiency. In addition to a compact design and fast response to changes in input power, PEMWE has a wider operating temperature range and is more economical in long-term applications [6,7,8].

However, the harsh corrosion of PEMWE in acidic environments makes most non-precious metal- and carbon-based catalysts unstable, making it impossible to achieve both high activity and durability. Therefore, acidic OER electrocatalysts still mainly rely on precious metals, such as iridium (Ir) and ruthenium (Ru) or their oxides. Ir has significant limitations in large-scale practical applications due to high cost, scarcity and other factors [9,10,11]. Ru costs only about 15% of the price of Ir, and is superior over Ir-based catalysts in terms of activity. Currently, RuO_2_ is one of the most effective catalysts demonstrated for OER at all pHs, making it a promising candidate for various applications [12,13,14,15]. However, its instability under highly corrosive and oxidizing acidic conditions still restricts its replacement of Ir-based catalysts and its large-scale application in PEMWE for hydrogen production [16,17,18].

Hence, it is necessary to further explore and develop high-activity and stable RuO_2_-based catalysts to improve the efficiency of water splitting under acidic conditions. Recently, researchers have proposed various strategies to enhance the activity and stability of RuO_2_-based catalysts, such as heterojunction engineering, heteroatom doping and coordination environment engineering. RuO_2_ doped with Ni, Sn doping or Ir, Sr co-doping is a much more efficient, durable and stable catalyst than commercial RuO_2_ for OER under acidic conditions [1,19,20]. The doping of these impurities enhances the adsorption of oxygen-containing intermediates on the Ru sites in OER, as well as the oxygen evolution activity, and reduces the reaction energy barrier of the rate-determining step. This method also effectively improves the charge transfer in RuO_2_, significantly weakens the covalency of Ru-O and completely suppresses the dissolution and over-oxidation of RuO_2_ during OER. Such improvements effectively solve the high catalyst cost and poor stability due to dissolution.

**Figure 1:**
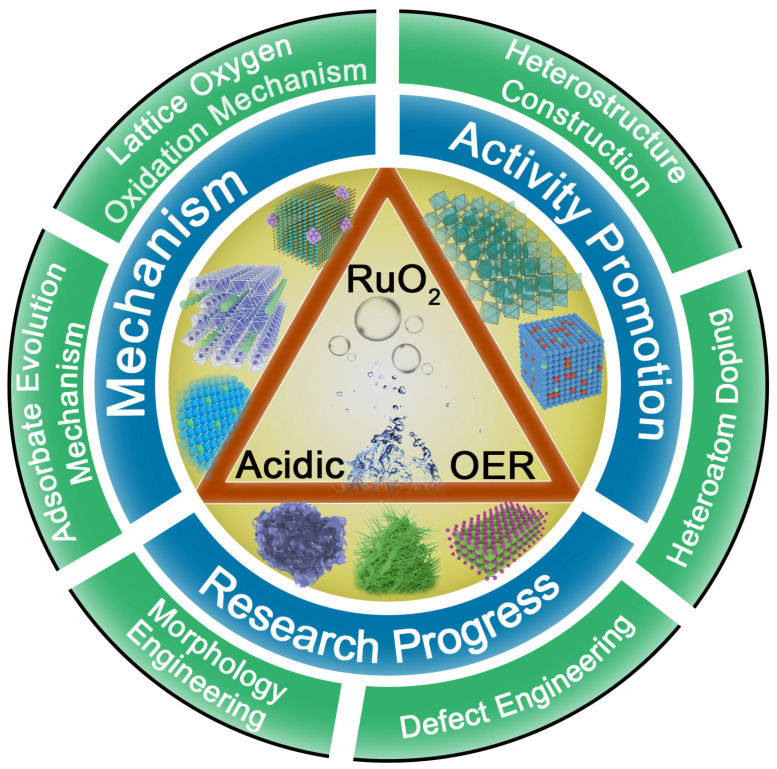
Scheme of the main content of RuO_2_ catalysts for electrocatalytic oxygen evolution in acidic media in this review.

In this article, we review the recent research progress on the OER of RuO_2_ electrocatalysts under acidic conditions, and summarize the common strategies to optimize and improve their OER catalytic activity and stability (Figure 1). We first discuss the two main mechanisms of OER under acidic conditions, providing guidance for modification design of RuO_2_ electrocatalysts and revealing the degradation/dissolution mechanism of RuO_2_ catalysts during OER. Finally, we summarize the current development prospects and challenges of RuO_2_-based electrocatalysts in acidic environments, and provide some reasonable solutions and suggestions for future research.

## 2. Reaction Mechanism

The OER pathways have a significant impact on the stability of catalysts in reactions, and they effectively lead to a deeper comprehension of the OER mechanism, as well as the rational design and preparation of catalysts. Ru-based catalysts often exhibit poor stability due to the formation of soluble Ru oxides (e.g., RuO_4_) during the OER process. RuO_2_-based OER materials often suffer from instability at high current density due to over-oxidation of Ru species (from Ru^0^ or Ru^4+^ to ortho-valent), and this over-oxidation of Ru is directly responsible for the stability [21]. In addition, for RuO_2_ electrocatalysts, the high energy barrier for the generation of the critical O* intermediate from OOH* as a rate-determining step (RDS) also has a great impact on the activity and durability of the catalyst [22]. Therefore, a detailed understanding of the OER reaction mechanism and a summary of relevant recent mechanistic studies will be of great help in enhancing the activity and stability of the catalysts. Typically, there are two potential reaction mechanisms: the traditional adsorbate evolution mechanism (AEM) and the lattice oxygen oxidation mechanism (LOM) [23,24,25].

### 2.1. Adsorbate Evolution Mechanism (AEM)

**Figure 2:**
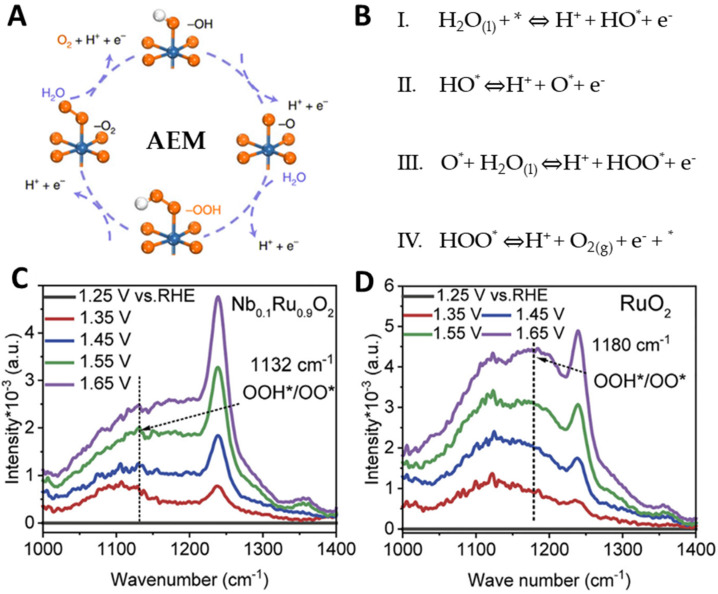
(**A**,**B**) Scheme of AEM pathway in acidic media, (**C**,**D**) ATR−SEIRAS analysis of Nb_0.1_Ru_0.9_O_2_ and RuO_2._ Copyright 2023, Cell Press.

AEM is a traditional OER mechanism, and its activity is highly correlated with the adsorption energy of the intermediate species. According to Sabatier’s principle, the accepted scaling relationships among various reaction intermediates highlight that the key factor influencing the reaction overpotential is the binding strength of the intermediates. Although the scaling relationships in AEM are helpful for efficient screening of catalysts, there are still significant limitations in improving OER activity [26,27]. The AEM pathway (Figure 2A,B) is typically believed to involve four synergistic proton-electron transfer reactions that occur at active metal sites during the OER process. The OER process involves three types of oxygen-containing adsorption intermediates, including oxygen radicals (O), hydroxyl groups (OH), and oxyhydroxide groups (OOH). First, the adsorbed water molecules lose electrons to form HO and generate O in the subsequent deprotonation step. Then, the O is subjected to nucleophilic attack by H_2_O molecules to form OOH, which is finally converted to O_2_ molecules and released in subsequent steps. In most OERs of the AEM pathway, the rate-determining step is typically considered to be the formation of HO or HOO*, and the difference in binding energy between HO* and HOO* is commonly used as a descriptor for OER activity. To achieve the optimal OER activity, the binding strength between reaction intermediates and the active sites should be set as moderate as possible to ensure the largest balance of adsorption and desorption energy. In the AEM pathway, the OER activity of the electrocatalysts is usually optimized by optimizing the binding strength of oxygen-containing intermediates and adjusting the electronic configuration of the catalyst.

Density functional theory (DFT) calculations of RuO_2_ electrocatalysts show that the interaction between O and the catalyst surface is too strong, inhibiting HOO* formation in the next step and limiting full catalytic activity [28,29]. Sun et al. captured HOO* in acidic OER using potential-dependent in situ attenuated total reflection Fourier transform infrared spectroscopy (ATR-SEIRAS), and studied the difference in binding energies of oxidized intermediates on the surface of the electrocatalysts (Figure 2C,D) [21]. At 1132 cm^−1^, the potential-dependent peak associated with stretching vibrations of OO/OOH became more pronounced when the potential shifted from 1.35 to 1.65 V. However, *OOH underwent stretching vibrations at 1.45 V for pure RuO_2_, with a blue shift to 1180 cm^−1^, indicating that the adsorption of *OOH on Nb_0.1_Ru_0.9_O_2_ is much stronger than that on RuO_2_.

### 2.2. Lattice Oxygen Oxidation Mechanism (LOM)

**Figure 3:**
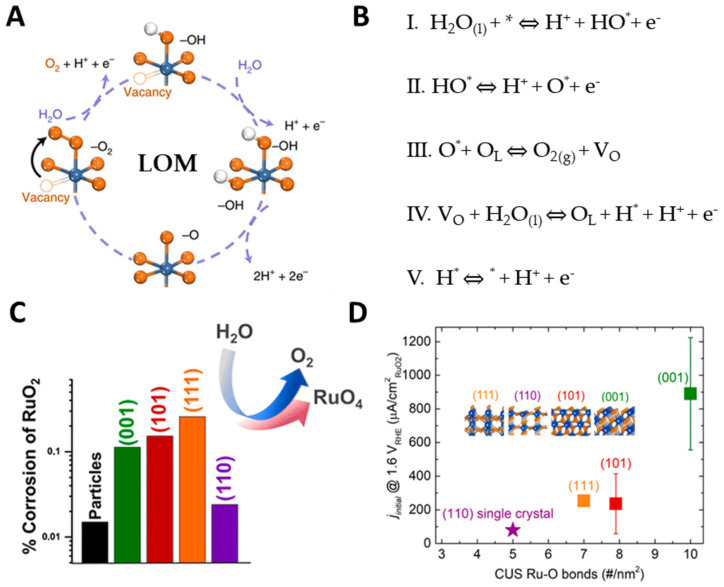
(**A**,**B**) Scheme of LOM pathway for acidic OER. (**C**) Histogram of the corrosion of RuO_2_ with (110), (111), (101), and (001) surface. (**D**) Current density and dissolution of RuO_2_. Copyright 2018, American Chemical Society.

The results of experiments and theoretical calculations suggest that a deeper understanding of the reaction mechanism that regulates catalyst activity predicts that oxygen evolution is closely related to the participation of lattice oxygen. LOM has been extensively studied to bypass the limitations of the AEM, showing that the active centers are not limited to metal centers [30]. LOM involves a non-coordinated proton-electron transfer step involving metal cationic active sites and lattice oxygen, involving five intermediate species. The first two reaction steps of the LOM pathway, involving the formation of O* and HO*, are similar to the AEM process (Figure 3A,B). The surface O* then combines with lattice oxygen in the catalyst structure to form a direct O-O bond. Then, through the formation of HO* and the removal of a proton through a single-electron oxidation step, a H_2_O molecule replenishes the surface vacancy. Therefore, the LOM bypasses the formation of OOH*, providing a different reaction path for direct coupling of lattice oxygen in the catalyst during OER. The limitation of the scale relationship between the intermediate free energy in LOM and the adsorption energy in AEM is quite different. Differential electrocatalytic mass spectrometry (DEMS) results reveal the involvement of lattice oxygen in OER under acidic conditions [31,32,33]. Lattice oxygen in RuO_2_ electrocatalysts participates in OER, easily forming soluble RuO_4_, which is a detectable corrosive product (Figure 3C,D) [34,35]. In contrast, no involvement of oxygen from platinum-based electrocatalysts was observed during OER [36]. Zagalskaya et al. used DFT to calculate the overpotential of the AEM and LOM pathways of the RuO_2_ catalyst, and explained the role of structural defects. Compared to catalysts with surface defects, catalysts with lattice oxygen vacancies have a higher overpotential [37]. For RuO_2_ electrocatalysts, the introduction of surface vacancies and dopants is likely to change the OER mechanism from AEM to LOM, confirming the susceptibility of RuO_2_ to structural defects.

Although LOM shows great potential in improving OER performance, the involvement of lattice oxygen in the reaction can easily lead to the instability of the catalyst due to thermodynamic expansion. The dissolution of cations and the continuous formation of oxygen vacancies during lattice oxygen oxidation-reduction, oxygen bulk diffusion and structural reconstruction occur, making the instability of LOM-based catalyst has become a key bottleneck in practical application. Therefore, in-depth elucidation of the LOM mechanism are crucial for the synthesis and development of high activity and high stability OER catalysts.

## 3. Activity and Stability Enhancement Strategies

RuO_2_-based catalysts (Table 1) are considered to be promising candidates for replacing iridium-based catalysts. However, the poor stability and corrosion resistance of ruthenium oxide under acidic conditions severely hinder its large-scale application as an OER electrocatalysts. Therefore, effective strategies are urgently needed to improve the activity and stability of Ru-based oxides for acidic water splitting [38,39,40,41,42]. Recently, various strategies are currently used to prepare modified acidic OER catalysts, such as heterostructure construction, atomic doping, defect engineering, and substrate engineering.

### 3.1. Heterostructure Construction

**Figure 4:**
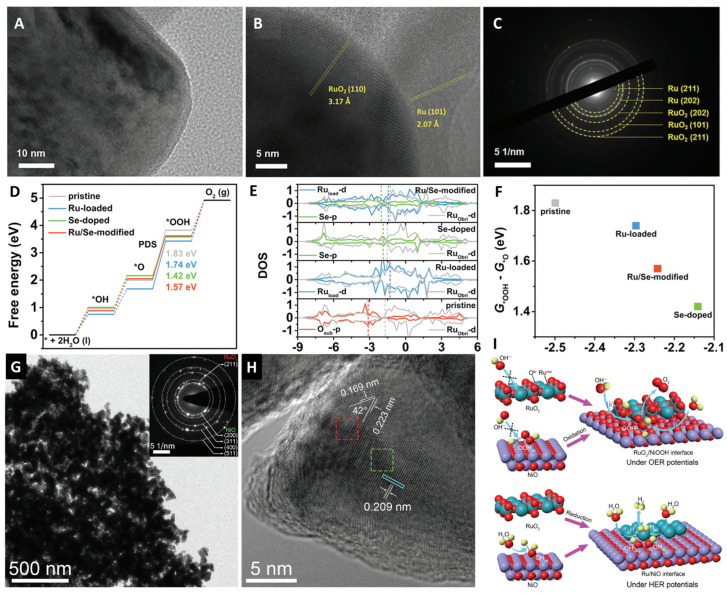
(**A**,**B**) HRTEM images and (**C**) SAED pattern of the Ru/Se-RuO_2_. (**D**) Free energy diagram, (**E**) DOS, (**F**) G_*OOH_-G_*O_ of all samples. Copyright 2022, Royal Society of Chemistry. (**G**) TEM image, (**H**) HRTEM image and (**I**) scheme of potential induced interfacial synergy of RuO_2_/NiO (red, O; yellow, H; cyan, Ru; purple, Ni). Copyright 2018, John Wiley & Sons.

The physical and chemical properties of metal materials can be further adjusted through various material synergy effects, optimized coordination environments, and electronic structures. Therefore, composite material design is an important way to construct high-activity acidic OER catalysts. After combining, different materials are combined with known efficient catalysts to form a heterostructure, the interfacial bonding interaction between different components can significantly enhance the electron transfer rate. The conductivity, hydrophilicity, chemical stability, and active site density of the heterostructure can be adjusted, resulting in easier access to catalytic active sites [52,55,56,57,58,59,60,61,62]. Different chemical compositions and crystal structures in heterostructures can cause lattice strain, which affects the adsorption energy of intermediates at the site, thereby enhancing the catalytic activity of the material. In heterostructures, the energy band arrangement of different phases may lead to interface charge transfer, which is beneficial for surface electronic modulation of heterostructures. The kinetic of electrocatalytic reactions can be improved by changing the composition and structure of the electrocatalyst at the molecular level [63,64,65,66,67,68,69]. Huang et al. synthesized a Ru/Se-RuO_2_ electrocatalyst through Se doping and Ru loading, and adjusted the phase composition and electronic structure of the Ru-based oxide [70]. The co-modification of Ru/Se on RuO_2_ reduced the adsorption free energy of *OOH intermediates, and enhanced the electronic transfer interaction and the formation of Ru/RuO_2_ heterojunctions, thus exhibiting excellent electrocatalytic performance in acidic OER with low overpotential and excellent long-term durability (Figure 4A–F).

**Figure 5:**
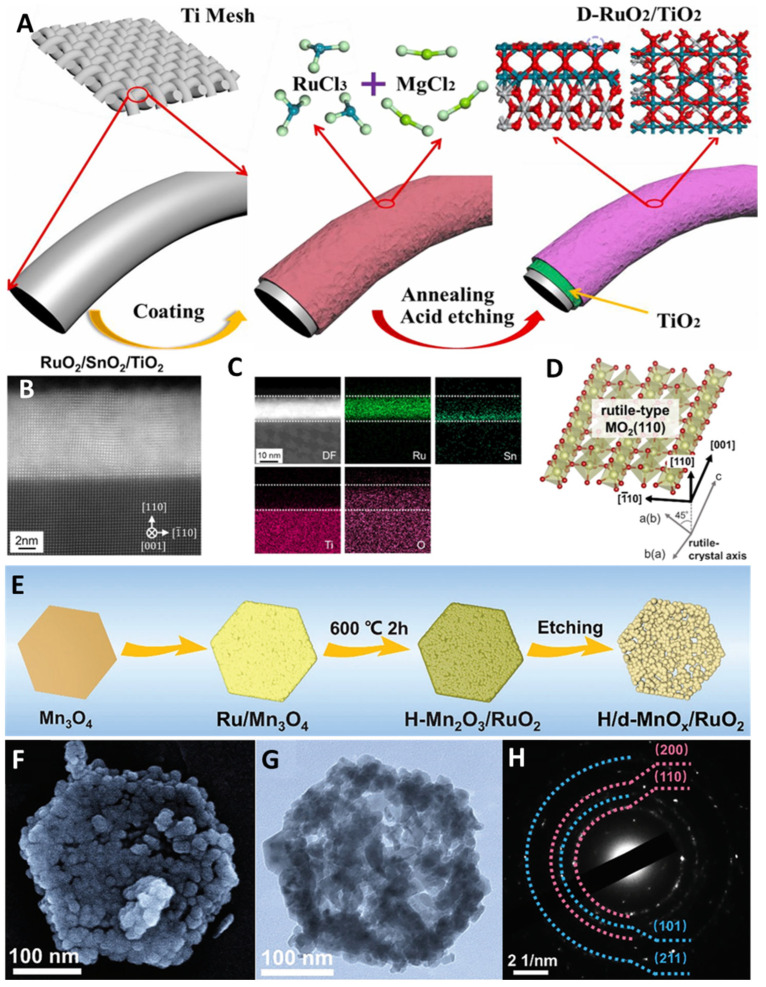
(**A**) Schematic fabrication of D-RuO_2_/TiO_2_ heterostructures. Copyright 2021 Elsevier Inc. (**B**) HAADF-STEM image and (**C**) STEM-EDS mappings of RuO_2_/SnO_2_/TiO_2_, (**D**) model of the rutile-RuO_2_ (110) surface. Copyright 2023 American Chemical Society. (**E**) Schematic illustration, (**F**) SEM, (**G**) TEM, and (**H**) SAED pattern of H/d-MnOx/RuO_2_. Copyright 2022, John Wiley & Sons.

Liu et al. designed a RuO_2_-NiO coupled electrocatalyst for OER, in which RuO_2_ nanoparticles were uniformly distributed on the surface of NiO [71]. This method provides a rich source of active sites and targeted interfacial synergy. The NiOOH derived from NiO enhanced the oxygen binding energy of RuO_2_, thereby improving the OER activity (Figure 4G–I). Li et al. synthesized a defect-based RuO_2_/TiO_2_ nanoheterostructure electrocatalyst (Figure 5A) [72]. This heterostructure can regulate the electronic structure of RuO_2_, the interface interaction between RuO_2_ and TiO_2_, and the defects on RuO_2_ nanoparticles, exposing a large number of active sites. The d-band center of Ru shifts to a lower energy level and weakens the interaction between adsorbed oxygen species on Ru sites, thereby enhancing catalytic activity. The TiO_2_ carrier with abundant oxygen vacancies significantly improves the OER activity and stability of the loaded RuO_2_-OER nanoparticles. Naoto Todoroki et al. introduced SnO_2_ to form a RuO_2_/Nb-TiO_2_ single-crystal oxide heterojunction catalyst (Figure 5B–D) [73]. The SnO_2_ interlayer stabilized the interface between RuO_2_ and TiO_2_ layers and thus reduced electrode resistance and lattice strains, inhibiting the formation of RuO_2_/TiO_2_ interface nanodomains and structural damage and alleviating the electrocatalytic and structural mismatch. As a result, the OER activity and stability were improved. Wu et al. designed a defect-rich MnOx/RuO_2_ nanosheet (H/d-MnOx/RuO_2_) [74]. The 2D hexagonal nanosheet shape fully exposes active sites (Figure 5E–H) and improves the utilization efficiency of precious metal atoms. Oxygen vacancies and non-homogeneous interfaces facilitate the reduction in *OOH adsorption energy and the Ru-O_ads_ energy level, inhibiting lattice oxygen participation, adjusting electronic structure of Ru, and accelerating electron transfer. Consequently, more electrons in the Ru-O_ads_ chemical bond become anti-bonding states, and the bond energy is reduced and the dissociation is promoted. As a result, the catalyst possesses high activity and durability.

### 3.2. Heteroatom Doping

**Figure 6:**
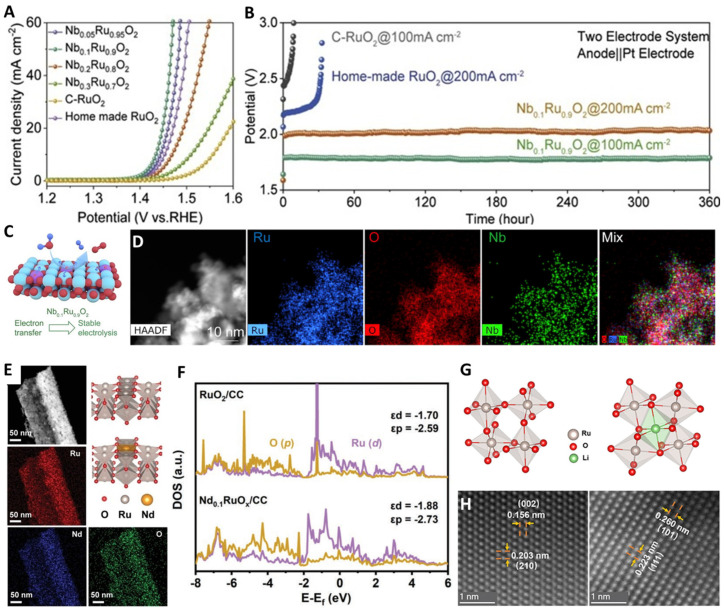
(**A**) Polarization curves, (**B**) chronopotentiometry tests, (**C**) structure diagram, (**D**) elemental mapping of Nb_0.1_Ru_0.9_O_2_. Copyright 2023, Cell press. (**E**) HRTEM image and crystal structure, (**F**) DOS of Nd−RuO_2_. Copyright 2023, John Wiley & Sons. (**G**) RuO_6_ octahedron before lithium intercalation and after lithium intercalation, (H) HAADF-STEM images of RuO_2_ (left) and Li_0.52_RuO_2_ (right). Copyright 2023, Springer Nature.

**Figure 7:**
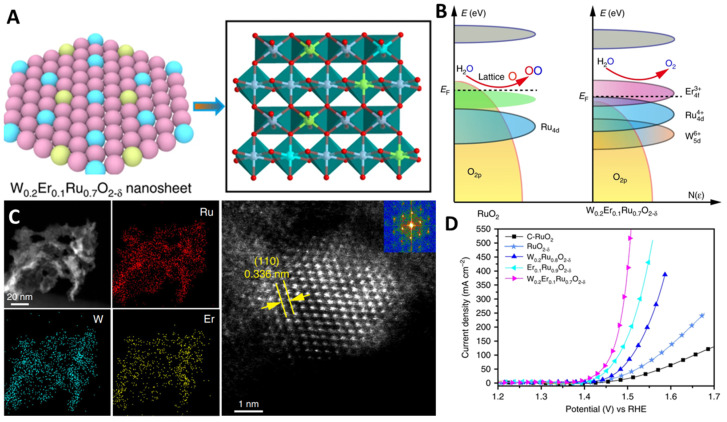
(**A**) Lattice oxygen oxidation way, (**B**) diagrams of rigid band models, (**C**) elemental maps and HRTEM image, (**D**) polarization curves of W_0.2_Er_0.1_Ru_0.7_O_2−δ_. Copyright 2020, Springer Nature.

In general, the formation or fracture of a bond depends on the bond strength between the oxygen intermediate and the active site, and the activity of a catalyst is greatly affected by its d-orbital electron configuration. The introduction of foreign atoms can effectively enhance the d-orbital state and electron transfer ability, and optimize the electron configuration of the catalyst, so as to promote the catalytic reaction. Cation doping has been widely studied to improve the intrinsic activity of Ru oxides, and various metallic elements (e.g., Ni, Nd, Nb, W, Er, Li, Mo, ln) can greatly improve the OER activity of Ru-based electrocatalysts in acidic media. Doping does not transform the OER mechanism, and the enhanced activity primarily stems from the optimized binding energy of oxygen intermediates [20,49,75,76,77,78,79,80,81,82,83,84,85,86,87,88,89,90,91,92,93]. Sun et al. synthesized Nb_0.1_Ru_0.9_O_2_ by doping high-valence refractory metal niobium into ruthenium oxide, which promoted the electron transfer in the local structure of Ru-O-Nb and reduced the valence state of Ru sites and the covalency of Ru-O bond [21]. Consequently, the adsorption of oxygen-containing intermediates on the Ru sites as well as oxygen evolution activity was enhanced. More importantly, the reaction energy barrier of the rate-determining step of ruthenium oxide was reduced, and excessive oxidation of the ruthenium sites and the participation of lattice oxygen in oxygen evolution were inhibited. Ultimately, the stability in oxygen evolution of ruthenium oxide was improved under high current density (Figure 6A–D). Li et al. used Nd-doped RuO_2_ (Nd_0.1_RuO_x_) as an efficient OER electrocatalysts for acidic solutions (Figure 6E,F) [43]. The high ratio of Ru^4+^ inhibited Ru dissolution in the acidic electrolyte, improving the stability of the electrocatalysts. The introduction of Nd reduced the d-band center energy of the electrocatalysts, effectively balanced the adsorption/desorption of oxygen-containing intermediates, and weakened the covalency of Ru-O, thus enhancing the catalytic activity. Qin et al. synthesized Li-doped RuO_2_ electrocatalysts by doping Li into the lattice of RuO_2_ (Figure 6G,H) [94]. The Ru valence was reduced with the formation of a stable Ru-O-Li, which weakened the covalent Ru-O and inhibited Ru dissolution, thereby improving its durability. Meanwhile, the intrinsic lattice strain caused by Li doping activated the dangling O atoms near the active Ru sites, stabilizing the intermediate OOH* and greatly enhancing its activity, and the overpotential was only 156 mV (@10 mA·cm^−2^). Hao et al. reported an advanced W_0.2_Er_0.1_Ru_0.7_O_2-δ_ electrocatalyst by introducing Er and W into RuO_2_ to change its electronic structure [44]. In addition, the over-oxidation and dissolution of Ru were effectively prevented, the active sites of Ru^4+^ in the acidic OER were maintained, and the formation of soluble RuO_4_ was inhibited. Moreover, the adsorption energy of oxygen-containing intermediates was reduced, and the energy required for the formation of oxygen vacancies was increased. Therefore, the overpotential was as low as 168 mV and the record-breaking stability was 500 h in acidic electrolytes (Figure 7).

Overall, the introduction of dopant atoms into the interstitial sites of the catalyst lattice to form doped compounds can adjust lattice parameters, ionic conductivity, and electronic structure [79]. Additionally, dopants can introduce different valence states by replacing lattice atoms, and adjust the energy levels and electron transfer abilities of the active sites. These atomic doping strategies provide valuable means for fine-tuning and optimizing OER reaction catalysts, ultimately enhancing catalytic performance and stability.

### 3.3. Defect Engineering

**Figure 8:**
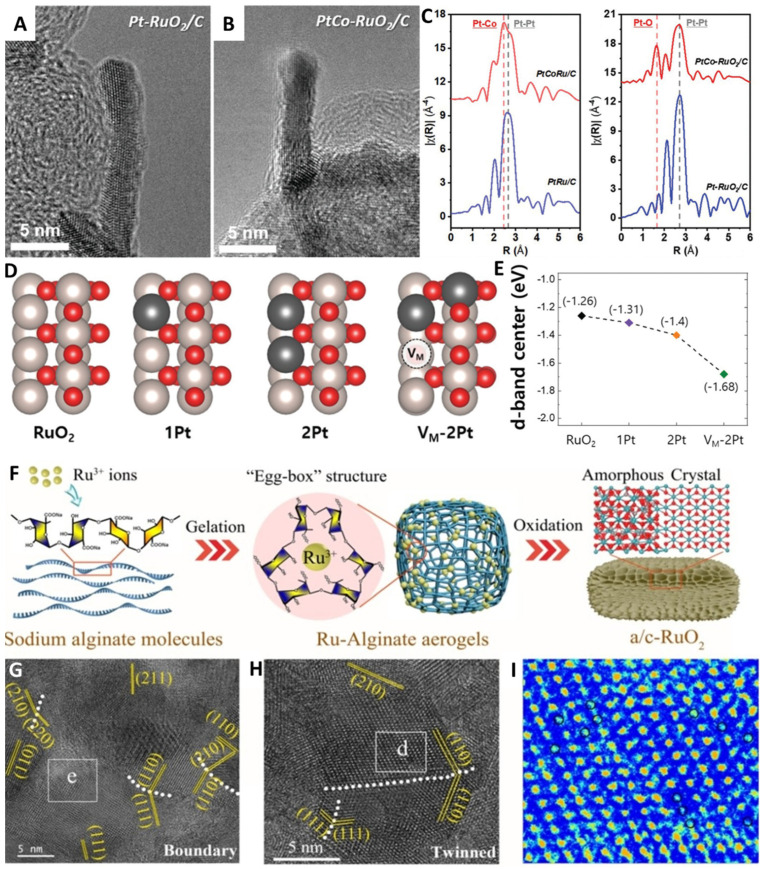
HRTEM images of (**A**,**B**) Pt–RuO_2_/C and PtCo–RuO_2_/C. (**C**) The FTEXAFS spectra of the Pt L3−edge. (**D**) The structures of bare and doped RuO_2_(110) slab. (**E**) The d-band center of Ru_cus_ in each case. Copyright 2022, John Wiley & Sons. (**F**) Scheme of synthesis process of a/c-RuO_2_. (**G**,**H**) HRTEM images of a/c-RuO_2_. (**I**) Filtered image of area. Copyright 2021, Royal Society of Chemistry.

Defect engineering involves the introduction or regulation of defect properties in a catalyst to alter its electronic structure, surface active sites, and proton transfer capability. This approach can enhance the activity and stability of OER. Additional active sites are provided and the interaction between the catalyst and oxygen molecules is strengthened by introducing defect sites (e.g., missing atoms, surface adsorbates, or oxygen vacancies) on the catalyst surface. The lattice structure and electronic transport properties can be altered by introducing defects into the catalyst lattice, such as vacancies, interstitial atoms, or substitutional atoms. These defects can influence the electronic structure of the catalyst and its ability to adsorb oxygen on its surface, thereby enhancing OER activity [15,94,95,96,97,98,99,100,101,102,103,104,105,106,107,108,109]. Defective RuO_2_ exhibits high initial activity, but can easily accelerate the dissolution of Ru species. Therefore, it is crucial to shift the electrocatalytic OER towards AEM to improve the durability of RuO_2_. As such, Jin et al. investigated the mixing of Pt atoms with a RuO_2_ matrix to obtain PtCo-RuO_2_/C with a nanorod shape [110]. The penetration of Pt into RuO_2_ and the dissolution of Co generated defects, which cooperatively reduced the d-band center of Ru, as well as the adsorption binding energy. Moreover, the adsorption and deprotonation of *OOH were promoted, the exposed Pt on the surface was oxidized, and electrons transferred from Pt to Ru, preventing the overoxidation of Ru and maintaining OER performance during long-term stability tests (Figure 8A–F). Zhang et al. reported a Na-doped amorphous/crystalline multi-phase RuO_2_ (a/c-RuO_2_) with oxygen vacancies, which served as an efficient OER electrocatalysts with significant acid and oxidation resistance, resulting in exceptional electrocatalytic stability [53]. Na doping and the introduction of oxygen vacancies lead to the deviation of the d band center of RuO_2_, which weakens the chemical bond between the oxygen-containing intermediates and the RuO_2_ surface, thus weakening the activation barrier of OER. The porous network structure of the catalyst exposed more active sites and facilitated the rapid transport of intermediates. Defects including vacancies (point defects) and boundaries (line defects) served as active sites and enhanced the reaction kinetics of OER (Figure 8G–I).

### 3.4. Morphology Engineering 

**Figure 9:**
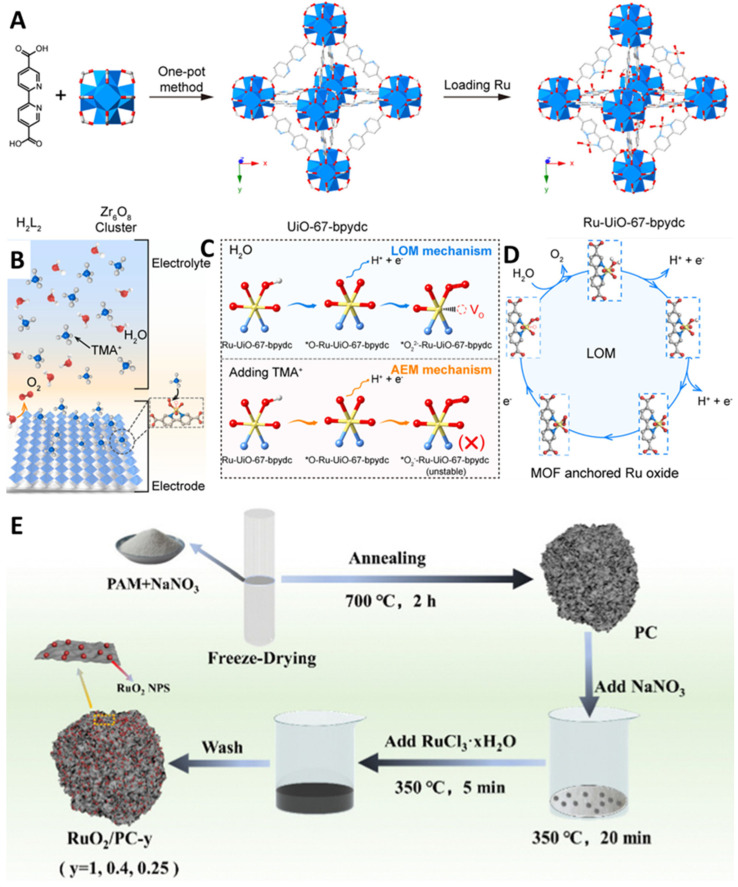
(**A**) Scheme of the fabricate of the Ru-UiO-67-bpydc. (**B**,**C**) the electrostatic interaction of the interface of Ru-UiO-67-bpydc. (**D**) LOM pathway of MOF-anchored Ru oxide toward the acidic OER. Copyright 2023, Cell Press. (**E**) Scheme of the preparation of RuO_2_/PC. Copyright 2023, Royal Society of Chemistry.

Morphology engineering is a promising way to expose more active sites or narrow the size of catalysts to the atomic, cluster, or nanoparticle scale and combine them with suitable carriers to enhance OER activity. The utilization efficiency of the active site can be significantly increased by the large surface area of the carrier carrying the active site. In addition, the coupling of metal particles or atoms with the carrier can induce significant electron transfer, which affects the adsorption of oxygen intermediates and thereby significantly enhances the activity and stability [111,112,113,114,115,116,117,118,119,120,121]. Although Ru-based oxide electrocatalysts exhibit high initial OER activity due to their kinetically favorable lattice oxygen oxidation mechanisms, the high-valence oxygen hole intermediate species are highly soluble, resulting in decreased catalytic activity and stability in acidic electrolytes. With a stable MOF modification strategy, Yao et al. reported a Ru-UiO-67-bpydc catalyst modified with atomically dispersed Ru oxide through pyridine coordination on the UiO-67 framework [122]. The Ru-N chemical bond not only enhanced the participation of lattice oxygen species in the OER, but also stabilized the intermediate species (*V_o_-${\mathrm{RuO}}_{4}^{2-}$), which also significantly reduced the position of the Ru d band center, increased the p band center position of O, promoted the LOM mechanism for OER, and stabilized the high-valence Ru intermediate species. Consequently, the OER performance and long-term stability of the electrocatalysts were greatly improved (Figure 9A–D). Yu et al. reported a hierarchical porous carbon-supported ruthenium dioxide (RuO_2_/PC) nanostructure (Figure 9E) [123]. The porous carbon provided a conductive platform for the RuO_2_ NPS, facilitating electron transfer and mass transport. It also offered larger electrocatalytic surface area to expose more active sites. With the support of PC, the acid resistance and structural stability of the RuO_2_/PC catalyst were further enhanced. The carbon phase and RuO_2_ lattice formed a rich heterointerface, which improved the electrocatalytic performance and stability of the RuO_2_ NPS.

## 4. Summary and Outlook

RuO_2_ is a promising OER electrocatalyst with high binding energy to oxygen intermediates. Its instability in OER is mainly due to over-oxidation and dissolution. This article reviews strategies (e.g., morphology engineering, doping, defect engineering, heterostructure engineering) that significantly optimize the stability and activity of RuO_2_ for electrocatalytic OER. On the one hand, unique morphology designing can provide rich active sites for oxygen-containing species. On the other hand, heterostructure engineering, defect engineering and effective electronic structure engineering can also improve the binding energy, thereby increasing the intrinsic activity. These advanced modification strategies are promising for applications in RuO_2_-based electrocatalysts.

Despite these achievements, this rapidly developing field still faces many challenges.

(1) Reaction mechanism. Understanding the correlation between the activity/stability and local structure is central to the design of efficient RuO_2_-based OER catalysts. The deactivation and dissolution of RuO_2_-based catalyst was studied by advanced technical means to provide guidance for the synthesis of more efficient OER electrocatalyst and the in-depth understanding of catalytic mechanism. Although in situ Raman and Operando XAS techniques can be used to record the oxidation states, geometry, electronic structures, and interfaces of catalysts, the diversity and complexity of active stages make it difficult to interpret the factors controlling and influencing catalytic reactions. In addition, the existing operando techniques can only capture quasi-stable active sites, while reaction intermediates typically have picosecond lifetime. Therefore, the development of more advanced techniques to study the electrocatalytic reaction process combined with theoretical simulation to better reveal the true and accurate electrochemical process will be of great help in improving the understanding of the acidic OER mechanism of RuO_2_-based catalyst.
(2) Activity and durability. Laboratories primarily use CV, CA, and CP to evaluate the stability of RuO_2_-based OER catalysts, but these methods ignore mass transport, electrode spacing, and fluid flow effects. Ru dissolution caused by over-oxidation in OER process is generally considered to be the main cause of deactivation of RuO_2_-based materials. And the stability degradation found in these validation methods can be caused not only by catalyst degradation, but also by catalyst interface separation or active site coverage. The development of accelerated deactivation test systems is necessary to provide information on long-term stability performance at high current densities and high temperatures that will be more useful for practical applications [124]. As applications are often required to operate at high current densities and high temperatures, accelerated deactivation test systems are required to rapidly assess catalyst degradation under these conditions. By accelerating the catalyst deactivation process, test times can be reduced, and the harsh environments of practical applications can be simulated, allowing catalyst stability data to be obtained more quickly, which can help researchers better understand the mechanisms of catalyst degradation and provide guidance on how to improve catalyst stability. In addition, the Accelerated Deactivation Test of RuO_2_-based catalyst can take into account important factors in practical applications, such as mass transfer, electrode spacing and fluid flow.
(3) Industrial applications. The small-scale durability under laboratory conditions cannot meet the requirements of industrial electrolytes. For large-scale PEM electrolysis applications, the catalyst shall be manufactured using scalable and industrially acceptable methods. Precise computer-aided 3D printing techniques can help construct complex structures, accelerating mass/charge/ion transport rates and enhancing activity and stability. Conductive substrates play a key role in delivering activity and stability, but common substrates are not stable in acids. By using acid oxidized and/or doped substrates, we can improve the corrosion resistance of the substrate, or an alternative substrate with excellent corrosion and oxidation resistance such as Ti or Ta foam could be an effective solution. Depositing conductive layers can inhibit the formation of insulating TiO_2_ layers to further enhance stability. The selection of appropriate OER substrate electrodes significantly impacts catalyst passivation/detachment, substrate-catalyst interactions, and stability performance. Testing in three-electrode configurations significantly differs from industrial applications in terms of operation conditions. To bridge the gap between material development and industrial applications, it is crucial to perform membrane electrode assembly (MEA) testing under relevant industrial conditions as early as possible and further understand the operational conditions and other components essential for designing an optimal working environment for MEAs.

In conclusion, RuO_2_-based materials show great potential for OER, but face many challenges. Nevertheless, with ongoing research and the emergence of new technologies, we have reason to believe that these issues will gradually be addressed. Meanwhile, we believe that there are enormous opportunities in applying high-performance OER catalysts into energy conversion technologies. Through interdisciplinary collaborations and joint efforts, we can look forward to further breakthroughs and progress in the future fields of energy conversion and storage.

## Figures and Tables

**Table 1:** Comparison of the overpotential at 10 mA cm^−2^ with the reported RuO_2_-based OER catalysts.

Sample	Overpotential @10 mA cm^−2^	Tafel Slope	Reference
Ni-RuO_2_	214	42.6	[1]
Ru@V-RuO_2_/C	176	45.6	[15]
Nb_0.1_Ru_0.9_O_2_	201	47.9	[21]
Mn_0.73_Ru_0.27_O_2−δ_	208	65.3	[22]
Nd_0.1_RuO_x_	211	50	[43]
W_0.2_Er_0.1_Ru_0.7_O_2−δ_	168	66.8	[44]
SS Pt RuO_2_ HNSs	228	51	[45]
In-RuO_2_/G	187	46.2	[46]
Bi_0.15_Ru_0.85_O_2_	200	59.6	[47]
La-RuO_2_	208	57.4	[48]
Re_0.06_Ru_0.94_O_2_	190	45.5	[49]
S-RuO_2_	219	54.2	[50]
Ru_0.6_Sn_0.4_O_2_	245	61.8	[51]
RuO_2_/CoOx	240	70	[52]
a/c-RuO_2_	205	48.6	[53]
Ru@RuO_2_	198	42.6	[54]

## Data Availability

Not applicable.

## References

[1] Wu Z.-Y., Chen F.-Y., Li B., Yu S.-W., Finfrock Y.Z., Meira D.M., Yan Q.-Q., Zhu P., Chen M.-X., Song T.-W. (2022). Non-iridium-based electrocatalyst for durable acidic oxygen evolution reaction in proton exchange membrane water electrolysis. Nat. Mater..

[2] Wang X., Xi S., Huang P., Du Y., Zhong H., Wang Q., Borgna A., Zhang Y.-W., Wang Z., Wang H. (2022). Pivotal role of reversible NiO_6_ geometric conversion in oxygen evolution. Nature.

[3] Yang Z., Chen H., Xiang M., Yu C., Hui J., Dong S. (2022). Coral reef structured cobalt-doped vanadate oxometalate nanoparticle for a high-performance electrocatalyst in water splitting. Int. J. Hydrogen Energy.

[4] Sun S.-C., Jiang H., Chen Z.-Y., Chen Q., Ma M.-Y., Zhen L., Song B., Xu C.-Y. (2022). Bifunctional WC-Supported RuO_2_ Nanoparticles for Robust Water Splitting in Acidic Media. Angew. Chem. Int. Ed..

[5] Sun H., Xu X., Kim H., Shao Z., Jung W. (2023). Advanced electrocatalysts with unusual active sites for electrochemical water splitting. InfoMat.

[6] Liu R.T., Xu Z.L., Li F.M., Chen F.Y., Yu J.Y., Yan Y., Chen Y., Xia B.Y. (2023). Recent advances in proton exchange membrane water electrolysis. Chem. Soc. Rev..

[7] Zhang K., Liang X., Wang L., Sun K., Wang Y., Xie Z., Wu Q., Bai X., Hamdy M.S., Chen H. (2022). Status and perspectives of key materials for PEM electrolyzer. Nano Res. Energy.

[8] Yuan S., Zhao C., Cai X., An L., Shen S., Yan X., Zhang J. (2023). Bubble evolution and transport in PEM water electrolysis: Mechanism, impact, and management. Prog. Energy Combust. Sci..

[9] Seitz L.C., Dickens C.F., Nishio K., Hikita Y., Montoya J., Doyle A., Kirk C., Vojvodic A., Hwang H.Y., Norskov J.K. (2016). A highly active and stable IrO_x_/SrIrO_3_ catalyst for the oxygen evolution reaction. Science.

[10] Wan G., Freeland J.W., Kloppenburg J., Petretto G., Nelson J.N., Kuo D.-Y., Sun C.-J., Wen J., Diulus J.T., Herman G.S. (2021). Amorphization mechanism of SrIrO_3_ electrocatalyst: How oxygen redox initiates ionic diffusion and structural reorganization. Sci. Adv..

[11] Lončar A., Escalera-López D., Cherevko S., Hodnik N. (2022). Inter-relationships between Oxygen Evolution and Iridium Dissolution Mechanisms. Angew. Chem. Int. Ed..

[12] Zhu F., Xue J., Zeng L., Shang J., Lu S., Cao X., Abrahams B.F., Gu H., Lang J. (2021). One-pot pyrolysis synthesis of highly active Ru/RuO_X_ nanoclusters for water splitting. Nano Res..

[13] Wang C., Jin L., Shang H., Xu H., Shiraishi Y., Du Y. (2021). Advances in engineering RuO_2_ electrocatalysts towards oxygen evolution reaction. Chin. Chem. Lett..

[14] Bai J., Cheng L., Liu S., Zhang H., Lian Y., Deng Y., Zhou Q., Tang Y., Su Y. (2024). Unravel the mechanism of d-orbital modulation and oxygen vacancy in cerium-doped RuO_2_ catalysts for acidic oxygen evolution reaction. Appl. Surf. Sci..

[15] Li Y., Wang W., Cheng M., Feng Y., Han X., Qian Q., Zhu Y., Zhang G. (2023). Arming Ru with Oxygen-Vacancy-Enriched RuO_2_ Sub-Nanometer Skin Activates Superior Bifunctionality for pH-Universal Overall Water Splitting. Adv. Mater..

[16] Wang T., Li Z., Jang H., Kim M.G., Qin Q., Liu X. (2023). Interface Engineering of Oxygen Vacancy-Enriched Ru/RuO_2_–Co_3_O_4_ Heterojunction for Efficient Oxygen Evolution Reaction in Acidic Media. ACS Sustain. Chem. Eng..

[17] Rao R.-R., Kolb M.J., Halck N.B., Pedersen A.F., Mehta A., You H., Stoerzinger K.A., Feng Z., Hansen H.A., Zhou H. (2017). Towards identifying the active sites on RuO_2_(110) in catalyzing oxygen evolution. Energy Environ. Sci..

[18] Lin Y., Dong Y., Wang X., Chen L. (2022). Electrocatalysts for the Oxygen Evolution Reaction in Acidic Media. Adv. Mater..

[19] Wen Y., Chen P., Wang L., Li S., Wang Z., Abed J., Mao X., Min Y., Dinh C.T., Luna P.D. (2021). Stabilizing Highly Active Ru Sites by Suppressing Lattice Oxygen Participation in Acidic Water Oxidation. J. Am. Chem. Soc..

[20] Shi Z., Li J., Wang Y., Liu S., Zhu J., Yang J., Wang X., Ni J., Jiang Z., Zhang L. (2023). Customized reaction route for ruthenium oxide towards stabilized water oxidation in high-performance PEM electrolyzers. Nat. Commun..

[21] Liu H., Zhang Z., Fang J., Li M., Sendeku M.G., Wang X., Wu H., Li Y., Ge J., Zhuang Z. (2023). Eliminating over-oxidation of ruthenium oxides by niobium for highly stable electrocatalytic oxygen evolution in acidic media. Joule.

[22] Wang K., Wang Y., Yang B., Li Z., Qin X., Zhang Q., Lei L., Qiu M., Wu G., Hou Y. (2022). Highly active ruthenium sites stabilized by modulating electron-feeding for sustainable acidic oxygen-evolution electrocatalysis. Energy Environ. Sci..

[23] Song J., Wei C., Huang Z.-F., Liu C., Zeng L., Wang X., Xu Z.-J. (2020). A review on fundamentals for designing oxygen evolution electrocatalysts. Chem. Soc. Rev..

[24] Zhu Y., Tahini H.A., Hu Z., Yin Y., Lin Q., Sun H., Zhong Y., Chen Y., Zhang F., Lin H.-J. (2020). Boosting oxygen evolution reaction by activation of lattice-oxygen sites in layered Ruddlesden-Popper oxide. EcoMat.

[25] Shan J., Zheng Y., Shi B., Davey K., Qiao S.-Z. (2019). Regulating Electrocatalysts via Surface and Interface Engineering for Acidic Water Electrooxidation. ACS Energy Lett..

[26] Vojvodic A., Nørskov J.K. (2011). Optimizing Perovskites for the Water-Splitting Reaction. Science.

[27] Hong W.-T., Risch M., Stoerzinger K.A., Grimaud A., Suntivich J., Shao-Horn Y. (2015). Toward the rational design of non-precious transition metal oxides for oxygen electrocatalysis. Energy Environ. Sci..

[28] Man I.C., Su H.-Y., Calle-Vallejo F., Hansen H.A., Martínez J.I., Inoglu N.G., Kitchin J., Jaramillo T.F., Nørskov J.K., Rossmeisl J. (2011). Universality in Oxygen Evolution Electrocatalysis on Oxide Surfaces. ChemCatChem.

[29] Wang C., Schechter A., Feng L. (2023). Iridium-based catalysts for oxygen evolution reaction in acidic media: Mechanism, catalytic promotion effects and recent progress. Nano Res. Energy.

[30] Damjanovic A., Jovanovic B. (1976). Anodic Oxide Films as Barriers to Charge Transfer in O_2_ Evolution at Pt in Acid Solutions. J. Electrochem. Soc..

[31] Grimaud A., Diaz-Morales O., Han B., Hong W.-T., Lee Y.-L., Giordano L., Stoerzinger K.A., Koper M.T.M., Shao-Horn Y. (2017). Activating lattice oxygen redox reactions in metal oxides to catalyse oxygen evolution. Nat. Chem..

[32] Willsau J., Wolter O., Heitbaum J. (1985). Does the oxide layer take part in the oxygen evolution reaction on platinum?: A DEMS study. J. Electroanal. Chem..

[33] Wohlfahrt-Mehrens M., Heitbaum J. (1987). Oxygen evolution on Ru and RuO_2_ electrodes studied using isotope labelling and on-line mass spectrometry. J. Electroanal. Chem..

[34] Kötz R., Stucki S., Scherson D., Kolb D.M. (1984). In-situ identification of RuO_4_ as the corrosion product during oxygen evolution on ruthenium in acid media. J. Electroanal. Chem..

[35] Roy C., Rao R.-R., Stoerzinger K.A., Hwang J., Rossmeisl J., Chorkendorff I., Shao-Horn Y., Stephens I.E.L. (2018). Trends in Activity and Dissolution on RuO_2_ under Oxygen Evolution Conditions: Particles versus Well-Defined Extended Surfaces. ACS Energy Lett..

[36] Cherevko S., Zeradjanin A.R., Topalov A.A., Kulyk N., Katsounaros I., Mayrhofer K.J.J. (2014). Dissolution of Noble Metals during Oxygen Evolution in Acidic Media. ChemCatChem.

[37] Zagalskaya A., Alexandrov V. (2020). Role of Defects in the Interplay between Adsorbate Evolving and Lattice Oxygen Mechanisms of the Oxygen Evolution Reaction in RuO_2_ and IrO_2_. ACS Catal..

[38] Jana J., Bhamu K.C., Ngo Y.-L.T., Kang S.G., Chung J.S., Hur S.H. (2021). Designing a bimetallic transition metal oxide/hydroxide composite for effective electrocatalytic oxygen evolution reaction. Appl. Surf. Sci..

[39] Chen M., Kitiphatpiboon N., Feng C., Abudula A., Ma Y., Guan G. (2023). Recent progress in transition-metal-oxide-based electrocatalysts for the oxygen evolution reaction in natural seawater splitting: A critical review. eScience.

[40] Feng Y., Yang H., Wang X., Hu C., Jing H., Cheng J. (2022). Role of transition metals in catalyst designs for oxygen evolution reaction: A comprehensive review. Int. J. Hydrogen Energy.

[41] Zhao C.-X., Liu J.-N., Wang J., Ren D., Li B.-Q., Zhang Q. (2021). Recent advances of noble-metal-free bifunctional oxygen reduction and evolution electrocatalysts. Chem. Soc. Rev..

[42] Liao F., Yin K., Ji Y., Zhu W., Fan Z., Li Y., Zhong J., Shao M., Kang Z., Shao Q. (2023). Iridium oxide nanoribbons with metastable monoclinic phase for highly efficient electrocatalytic oxygen evolution. Nat. Commun..

[43] Li L., Zhang G., Xu J., He H., Wang B., Yang Z., Yang S. (2023). Optimizing the Electronic Structure of Ruthenium Oxide by Neodymium Doping for Enhanced Acidic Oxygen Evolution Catalysis. Adv. Funct. Mater..

[44] Hao S., Liu M., Pan J., Liu X., Tan X., Xu N., He Y., Lei L., Zhang X. (2020). Dopants fixation of Ruthenium for boosting acidic oxygen evolution stability and activity. Nat. Commun..

[45] Wang J., Yang H., Li F., Li L., Wu J., Liu S., Cheng T., Xu Y., Shao Q., Huang X. (2022). Single-site Pt-doped RuO_2_ hollow nanospheres with interstitial C for high-performance acidic overall water splitting. Sci. Adv..

[46] Wang Y., Lei X., Zhang B., Bai B., Das P., Azam T., Xiao J., Wu Z.S. (2023). Breaking the Ru-O-Ru Symmetry of a RuO_2_ Catalyst for Sustainable Acidic Water Oxidation. Angew. Chem. Int. Ed. Engl..

[47] Wu L., Liang Q., Zhao J., Zhu J., Jia H., Zhang W., Cai P., Luo W. (2023). A Bi-doped RuO_2_ catalyst for efficient and durable acidic water oxidation. Chin. J. Catal..

[48] Wu Y., Yao R., Zhao Q., Li J., Liu G. (2022). La-RuO_2_ nanocrystals with efficient electrocatalytic activity for overall water splitting in acidic media: Synergistic effect of La doping and oxygen vacancy. Chem. Eng. J..

[49] Jin H., Liu X., An P., Tang C., Yu H., Zhang Q., Peng H.J., Gu L., Zheng Y., Song T. (2023). Dynamic rhenium dopant boosts ruthenium oxide for durable oxygen evolution. Nat. Commun..

[50] Liu L., Ji Y., You W., Liu S., Shao Q., Kong Q., Hu Z., Tao H., Bu L., Huang X. (2023). Trace Lattice S Inserted RuO_2_ Flexible Nanosheets for Efficient and Long-Term Acidic Oxygen Evolution Catalysis. Small.

[51] Jia S., Zhang J., Liu Q., Ma C., Tang Y., Sun H. (2023). Competitive adsorption of oxygen-containing intermediates on ruthenium–tin solid-solution oxides for alkaline oxygen evolution. J. Mater. Chem. A.

[52] Du K., Zhang L., Shan J., Guo J., Mao J., Yang C.C., Wang C.H., Hu Z., Ling T. (2022). Interface engineering breaks both stability and activity limits of RuO_2_ for sustainable water oxidation. Nat. Commun..

[53] Zhang L., Jang H., Liu H., Kim M.G., Yang D., Liu S., Liu X., Cho J. (2021). Sodium-Decorated Amorphous/Crystalline RuO_2_ with Rich Oxygen Vacancies: A Robust pH-Universal Oxygen Evolution Electrocatalyst. Angew. Chem. Int. Ed. Engl..

[54] Li Z., Zou J., Liang T., Song X., Li Z., Wen J., Peng M., Zeng X., Huang H., Wu H. (2023). MOF-derived ultrasmall Ru@RuO_2_ heterostructures as bifunctional and pH-universal electrocatalysts for 0.79 V asymmetric amphoteric overall water splitting. Chem. Eng. J..

[55] Li W., Zhao L., Wang C., Lu X., Chen W. (2021). Interface Engineering of Heterogeneous CeO_2_-CoO Nanofibers with Rich Oxygen Vacancies for Enhanced Electrocatalytic Oxygen Evolution Performance. ACS Appl. Mater. Interfaces.

[56] Zeng L., Mao G., Zhu Y., Li R., Zhou Q., Xiao F., Tang R. (2022). Accelerated oxygen evolution enabled by encapsulating hybrid CoO_x_/RuO_2_ nanoparticle with nanoporous carbon. Appl. Surf. Sci..

[57] Ma Z., Zhang Y., Liu S., Xu W., Wu L., Hsieh Y.C., Liu P., Zhu Y., Sasaki K., Renner J.N. (2018). Reaction mechanism for oxygen evolution on RuO_2_, IrO_2_, and RuO_2_@IrO_2_ core-shell nanocatalysts. Electroanal. Chem..

[58] Lin C., Li J.-L., Li X., Yang S., Luo W., Zhang Y., Kim S.H., Kim D.H., Shinde S.S., Li Y.-F. (2021). In-situ reconstructed Ru atom array on α-MnO_2_ with enhanced performance for acidic water oxidation. Nat. Catal..

[59] Wang J., Kim H., Lee H., Ko Y.J., Han M.-H., Kim W., Baik J.M., Choi J.Y., Oh H.S., Lee W.H. (2023). Sb incorporated into oxides enhances stability in acid during the oxygen evolution reaction by inhibiting structural distortion. Nano Energy.

[60] Hu T., Wang Y., Zhang L., Tang T., Xiao H., Chen W., Zhao M., Jia J., Zhu H. (2019). Facile synthesis of PdO-doped Co_3_O_4_ nanoparticles as an efficient bifunctional oxygen electrocatalyst. Appl. Catal. B—Environ..

[61] Hou L., Li Z., Jang H., Wang Y., Cui X., Gu X., Kim M.G., Feng L., Liu S., Liu X. (2023). Electronic and Lattice Engineering of Ruthenium Oxide towards Highly Active and Stable Water Splitting. Adv. Energy Mater..

[62] Xu J., Chen C., Kong X. (2023). Ru-O-Cu center constructed by catalytic growth of Ru for efficient hydrogen evolution. Nano Energy.

[63] Lu X., Yang Y., Yin Y., Wang Z., Sutrisno L., Yan C., Schmidt O.G. (2021). Atomic Heterointerface Boosts the Catalytic Activity toward Oxygen Reduction/Evolution Reaction. Adv. Energy Mater..

[64] Duan Y., Sun S., Sun Y., Xi S., Chi X., Zhang Q., Ren X., Wang J., Ong S.J.H., Du Y. (2019). Mastering Surface Reconstruction of Metastable Spinel Oxides for Better Water Oxidation. Adv. Mater..

[65] Wang T., Wang P., Zang W., Li X., Chen D., Kou Z., Mu S., Wang J. (2021). Nanoframes of Co_3_O_4_–Mo_2_N Heterointerfaces Enable High-Performance Bifunctionality toward Both Electrocatalytic HER and OER. Adv. Funct. Mater..

[66] Huang J., Sheng H., Ross R.D., Han J., Wang X., Song B., Jin S. (2021). Modifying redox properties and local bonding of Co_3_O_4_ by CeO_2_ enhances oxygen evolution catalysis in acid. Nat. Commun..

[67] Wang X., Yu L., Guan B.-Y., Song S., Lou X.W.D. (2018). Metal-Organic Framework Hybrid-Assisted Formation of Co_3_O_4_/Co-Fe Oxide Double-Shelled Nanoboxes for Enhanced Oxygen Evolution. Adv. Mater..

[68] Zhang X., Feng C., Dong B., Liu C., Chai Y. (2023). High-Voltage-Enabled Stable Cobalt Species Deposition on MnO_2_ for Water Oxidation in Acid. Adv. Mater..

[69] Huang B., Cui Y., Liu X., Zheng C., Wang H., Guan L. (2023). Dense-Packed RuO_2_ Nanorods with In Situ Generated Metal Vacancies Loaded on SnO_2_ Nanocubes for Proton Exchange Membrane Water Electrolyzer with Ultra-Low Noble Metal Loading. Small.

[70] Huang K., Lin C., Yu G., Du P., Xie X., He X., Zheng Z., Sun N., Tang H., Li X. (2022). Ru/Se-RuO_2_ Composites via Controlled Selenization Strategy for Enhanced Acidic Oxygen Evolution. Adv. Funct. Mater..

[71] Liu J., Zheng Y., Jiao Y., Wang Z., Lu Z., Vasileff A., Qiao S.-Z. (2018). NiO as a Bifunctional Promoter for RuO_2_ toward Superior Overall Water Splitting. Small.

[72] Li W., Zhang H., Hong M., Zhang L., Feng X., Shi M., Hu W., Mu S. (2022). Defective RuO_2_/TiO_2_ nano-heterostructure advances hydrogen production by electrochemical water splitting. Chem. Eng. J..

[73] Todoroki N., Kudo R., Hayashi K., Yokoi M., Naraki N., Wadayama T. (2023). Improving the Oxygen Evolution Activity and Stability of Nb-Doped TiO_2_-Supported RuO_2_ by a SnO_2_ Interlayer: A Model Catalyst Study on Single-Crystal Oxide Heterostructures. ACS Catal..

[74] Wu Z., Wang Y., Liu D., Zhou B., Yang P., Liu R., Xiao W., Ma T., Wang J., Wang L. (2023). Hexagonal Defect-Rich MnO_x_/RuO_2_ with Abundant Heterointerface to Modulate Electronic Structure for Acidic Oxygen Evolution Reaction. Adv. Funct. Mater..

[75] Banerjee S., Debata S., Madhuri R., Sharma P.K. (2018). Electrocatalytic behavior of transition metal (Ni, Fe, Cr) doped metal oxide nanocomposites for oxygen evolution reaction. Appl. Surf. Sci..

[76] Al-Naggar A.H., Shinde N.M., Kim J.-S., Mane R.S. (2023). Water splitting performance of metal and non-metal-doped transition metal oxide electrocatalysts. Coord. Chem. Rev..

[77] Wang N., Ou P., Miao R.-K., Chang Y., Wang Z., Hung S.-F., Abed J., Ozden A., Chen H.-Y., Wu H.-L. (2023). Doping Shortens the Metal/Metal Distance and Promotes OH Coverage in Non-Noble Acidic Oxygen Evolution Reaction Catalysts. J. Am. Chem. Soc..

[78] Peng Y., Hajiyani H., Pentcheva R. (2021). Influence of Fe and Ni Doping on the OER Performance at the Co_3_O_4_(001) Surface: Insights from DFT+U Calculations. ACS Catal..

[79] Ko Y.-J., Han M.-H., Lim C., Yu S.-H., Choi C.H., Min B.K., Choi J.Y., Lee W.H., Oh H.-S. (2023). Unveiling the role of Ni in Ru-Ni oxide for oxygen evolution: Lattice oxygen participation enhanced by structural distortion. J. Energy Chem..

[80] Deng M., Tang Y., Lu Z., Wang Y., Lin Y. (2023). Self-Supporting Mn-RuO2 Nanoarrays for Stable Oxygen Evolution Reaction in Acid. Molecules.

[81] Zhang S.-L., Guan B.-Y., Lu X.-F., Xi S., Du Y., Lou X.W.D. (2020). Metal Atom-Doped Co_3_O_4_ Hierarchical Nanoplates for Electrocatalytic Oxygen Evolution. Adv. Mater..

[82] Huang Y., Li M., Pan F., Zhu Z., Sun H., Tang Y., Fu G. (2022). Plasma-induced Mo-doped Co_3_O_4_ with enriched oxygen vacancies for electrocatalytic oxygen evolution in water splitting. Carbon Energy.

[83] Zhu Y., Wang J., Koketsu T., Kroschel M., Chen J.-M., Hsu S.Y., Henkelman G., Hu Z., Strasser P., Ma J. (2022). Iridium single atoms incorporated in Co_3_O_4_ efficiently catalyze the oxygen evolution in acidic conditions. Nat. Commun..

[84] Liu M., Ji Y., Li Y., An P., Zhang J., Yan J., Liu S.-F. (2021). Single-Atom Doping and High-Valence State for Synergistic Enhancement of NiO Electrocatalytic Water Oxidation. Small.

[85] Liu Y., Ye C., Zhao S.-N., Wu Y., Liu C., Huang J., Xue L., Sun J., Zhang W., Wang X. (2022). A dual-site doping strategy for developing efficient perovskite oxide electrocatalysts towards oxygen evolution reaction. Nano Energy.

[86] An L., Zhang H., Zhu J., Xi S., Huang B., Sun M., Peng Y., Xi P., Yan C.H. (2023). Balancing Activity and Stability in Spinel Cobalt Oxides through Geometrical Sites Occupation towards Efficient Electrocatalytic Oxygen Evolution. Angew. Chem. Int. Ed..

[87] Li L., Cao X., Huo J., Qu J., Chen W., Liu C., Zhao Y., Liu H., Wang G. (2023). High valence metals engineering strategies of Fe/Co/Ni-based catalysts for boosted OER electrocatalysis. J. Energy Chem..

[88] Zhao J., He Y., Wang J., Zhang J., Qiu L., Chen Y., Zhong C., Han X., Deng Y., Hu W. (2022). Regulating metal active sites of atomically-thin nickel-doped spinel cobalt oxide toward enhanced oxygen electrocatalysis. Chem. Eng. J..

[89] Yu J., Garces-Pineda F.A., Gonzalez-Cobos J., Pena-Diaz M., Rogero C., Gimenez S., Spadaro M.C., Arbiol J., Barja S., Galan-Mascaros J.R. (2022). Sustainable oxygen evolution electrocatalysis in aqueous 1 M H_2_SO_4_ with earth abundant nanostructured Co_3_O_4_. Nat. Commun..

[90] Zhang N., Hu Y., An L., Li Q., Yin J., Li J., Yang R., Lu M., Zhang S., Xi P. (2022). Surface Activation and Ni-S Stabilization in NiO/NiS_2_ for Efficient Oxygen Evolution Reaction. Angew. Chem. Int. Ed..

[91] Chen S., Huang H., Jiang P., Yang K., Diao J., Gong S., Liu S., Huang M., Wang H., Chen Q. (2019). Mn-Doped RuO_2_ Nanocrystals as Highly Active Electrocatalysts for Enhanced Oxygen Evolution in Acidic Media. ACS Catal..

[92] Wang Y., Yang R., Ding Y., Zhang B., Li H., Bai B., Li M., Cui Y., Xiao J., Wu Z.S. (2023). Unraveling oxygen vacancy site mechanism of Rh-doped RuO_2_ catalyst for long-lasting acidic water oxidation. Nat. Commun..

[93] Qiu L., Zheng G., He Y., Lei L., Zhang X. (2021). Ultra-small Sn-RuO_2_ nanoparticles supported on N-doped carbon polyhedra for highly active and durable oxygen evolution reaction in acidic media. Chem. Eng. J..

[94] Qin Y., Yu T., Deng S., Zhou X.-Y., Lin D., Zhang Q., Jin Z., Zhang D., He Y.-B., Qiu H.-J. (2022). RuO_2_ electronic structure and lattice strain dual engineering for enhanced acidic oxygen evolution reaction performance. Nat. Commun..

[95] Li Y., Wang Y., Lu J., Yang B., San X., Wu Z.-S. (2020). 2D intrinsically defective RuO_2_/Graphene heterostructures as All-pH efficient oxygen evolving electrocatalysts with unprecedented activity. Nano Energy.

[96] Ge R.-X., Li L., Su J.-W., Lin Y.-C., Tian Z.-Q., Chen L. (2019). Ultrafine Defective RuO_2_ Electrocatayst Integrated on Carbon Cloth for Robust Water Oxidation in Acidic Media. Adv. Energy Mater..

[97] Adegoke K.A., Maxakato N.W. (2022). Porous metal oxide electrocatalytic nanomaterials for energy conversion: Oxygen defects and selection techniques. Coord. Chem. Rev..

[98] Zhang R., Pan L., Guo B., Huang Z.-F., Chen Z., Wang L., Zhang X., Guo Z., Xu W., Loh K.P. (2023). Tracking the Role of Defect Types in Co_3_O_4_ Structural Evolution and Active Motifs during Oxygen Evolution Reaction. J. Am. Chem. Soc..

[99] Béjar J., Álvarez-Contreras L., Guerra-Balcázar M., Ledesma-García J., Arriaga L.G., Arjona N. (2020). Synthesis of a small-size metal oxide mixture based on MoO and NiO with oxygen vacancies as bifunctional electrocatalyst for oxygen reactions. Appl. Surf. Sci..

[100] Yao Q., Huang B., Xu Y., Li L., Shao Q., Huang X. (2021). A chemical etching strategy to improve and stabilize RuO_2_-based nanoassemblies for acidic oxygen evolution. Nano Energy.

[101] Xiao Z., Huang Y.C., Dong C.L., Xie C., Liu Z., Du S., Chen W., Yan D., Tao L., Shu Z. (2020). Operando Identification of the Dynamic Behavior of Oxygen Vacancy-Rich Co_3_O_4_ for Oxygen Evolution Reaction. J. Am. Chem. Soc..

[102] Zeng H., Oubla M.h., Zhong X., Alonso-Vante N., Du F., Xie Y., Huang Y., Ma J. (2021). Rational defect and anion chemistries in Co_3_O_4_ for enhanced oxygen evolution reaction. Appl. Catal. B—Environ..

[103] Xu L., Jiang Q., Xiao Z., Li X., Huo J., Wang S., Dai L. (2016). Plasma-Engraved Co_3_O_4_ Nanosheets with Oxygen Vacancies and High Surface Area for the Oxygen Evolution Reaction. Angew. Chem. Int. Ed. Engl..

[104] Tian Y., Liu X., Xu L., Yuan D., Dou Y., Qiu J., Li H., Ma J., Wang Y., Su D. (2021). Engineering Crystallinity and Oxygen Vacancies of Co(II) Oxide Nanosheets for High Performance and Robust Rechargeable Zn–Air Batteries. Adv. Funct. Mater..

[105] Chen J., Cui P., Zhao G., Rui K., Lao M., Chen Y., Zheng X., Jiang Y., Pan H., Dou S.-X. (2019). Low–Coordinate Iridium Oxide Confined on Graphitic Carbon Nitride for Highly Efficient Oxygen Evolution. Angew. Chem. Int. Ed..

[106] Zhang J., Qian J., Ran J., Xi P., Yang L., Gao D. (2020). Engineering Lower Coordination Atoms onto NiO/Co_3_O_4_ Heterointerfaces for Boosting Oxygen Evolution Reactions. ACS Catal..

[107] Nong H.-N., Reier T., Oh H.S., Gliech M., Paciok P., Vu T.H.T., Teschner D., Heggen M., Petkov V., Schlögl R. (2018). A unique oxygen ligand environment facilitates water oxidation in hole-doped IrNiO_x_ core–shell electrocatalysts. Nat. Catal..

[108] Wang Z., Wu T., Zhu K., Xie W., Zhu X., Yang W. (2023). Interface modulation of perovskite oxides to simultaneously enhance the activity and stability toward oxygen evolution reaction. Chem. Eng. J..

[109] Yan H., Jiang Z., Deng B., Wang Y., Jiang Z.-J. (2023). Ultrathin Carbon Coating and Defect Engineering Promote RuO_2_ as an Efficient Catalyst for Acidic Oxygen Evolution Reaction with Super-High Durability. Adv. Energy Mater..

[110] Jin H., Choi S., Bang G.-J., Kwon T., Kim H.S., Lee S.J., Hong Y., Lee D.W., Park H.S., Baik H. (2022). Safeguarding the RuO_2_ phase against lattice oxygen oxidation during acidic water electrooxidation. Energy Environ. Sci..

[111] Liao C., Yang B., Zhang N., Liu M., Chen G., Jiang X., Chen G., Yang J., Liu X., Chan T.-S. (2019). Constructing Conductive Interfaces between Nickel Oxide Nanocrystals and Polymer Carbon Nitride for Efficient Electrocatalytic Oxygen Evolution Reaction. Adv. Funct. Mater..

[112] Ruiz-Cornejo J.C., Vivo-Vilches J.F., Sebastián D., Martínez-Huerta M.V., Lázaro M.J. (2021). Carbon nanofiber-supported tantalum oxides as durable catalyst for the oxygen evolution reaction in alkaline media. Renew. Energy.

[113] Chalgin A., Song C., Tao P., Shang W., Deng T., Wu J. (2020). Effect of supporting materials on the electrocatalytic activity, stability and selectivity of noble metal-based catalysts for oxygen reduction and hydrogen evolution reactions. Prog. Nat. Sci..

[114] Kumar P., Kannimuthu K., Zeraati A.S., Roy S., Wang X., Wang X., Samanta S., Miller K.A., Molina M., Trivedi D. (2023). High-Density Cobalt Single-Atom Catalysts for Enhanced Oxygen Evolution Reaction. J. Am. Chem. Soc..

[115] Meng L., Liu W., Lu Y., Liang Z., He T., Li J., Nan H., Luo S., Yu J. (2023). Lamellar-stacked cobalt-based nanopiles integrated with nitrogen/sulfur co-doped graphene as a bifunctional electrocatalyst for ultralong-term zinc-air batteries. J. Energy Chem..

[116] Ruiz-Cornejo J.C., Sebastián D., Pardo J.I., Martínez-Huerta M.V., Lázaro M.J. (2022). Sulfur-doped carbon nanofibers as support for tantalum oxides bifunctional catalysts for the oxygen reduction and evolution reactions. J. Power Sources.

[117] Browne M.P., Tyndall D., Nicolosi V. (2022). The potential of MXene materials as a component in the catalyst layer for the Oxygen Evolution Reaction. Curr. Opin. Electrochem..

[118] Yang X., Chen J., Chen Y., Feng P., Lai H., Li J., Luo X. (2018). Novel Co_3_O_4_ Nanoparticles/Nitrogen-Doped Carbon Composites with Extraordinary Catalytic Activity for Oxygen Evolution Reaction (OER). Nano-Micro Lett..

[119] Zhang B., Luo Y., Xiang D., Qin J., Miao K., Wang X., Kang X., Tian Y. (2023). Yolk-Shell Structured Zinc-Cobalt-Ruthenium Alloy Oxide Assembled with Ultra-Small Nanoparticles: A Superior Cascade Catalyst toward Oxygen Evolution Reaction. Adv. Funct. Mater..

[120] Du J., Chen D., Ding Y., Wang L., Li F., Sun L. (2023). Highly Stable and Efficient Oxygen Evolution Electrocatalyst Based on Co Oxides Decorated with Ultrafine Ru Nanoclusters. Small.

[121] Jiao H., Wang C., Zhang Z.-Y., Song Y.-F., Feng B.-Q., Na P., Wang Z.-L. (2023). Ultrafine NiFe-Based (Oxy)Hydroxide Nanosheet Arrays with Rich Edge Planes and Superhydrophilic-Superaerophobic Characteristics for Oxygen Evolution Reaction. Small.

[122] Yao N., Jia H., Zhu J., Shi Z., Cong H., Ge J., Luo W. (2023). Atomically dispersed Ru oxide catalyst with lattice oxygen participation for efficient acidic water oxidation. Chem.

[123] Yu B., Liu J.-H., Guo S., Huang G., Zhang S., Chen S., Li X., Wang Y., Lv L.P. (2023). Densely populated tiny RuO_2_ crystallites supported by hierarchically porous carbon for full acidic water splitting. Mater. Horiz..

[124] Tang J., Xu X., Tang T., Zhong Y., Shao Z. (2022). Perovskite-Based Electrocatalysts for Cost-Effective Ultrahigh-Current-Density Water Splitting in Anion Exchange Membrane Electrolyzer Cell. Small Methods.

